# MRI-visible perivascular spaces are associated with cerebrospinal fluid biomarkers in Parkinson’s disease

**DOI:** 10.18632/aging.104200

**Published:** 2020-11-25

**Authors:** Yi Fang, Lu-Yan Gu, Jun Tian, Shao-Bing Dai, Ying Chen, Ran Zheng, Xiao-Li Si, Chong-Yao Jin, Zhe Song, Ya-Ping Yan, Xin-Zhen Yin, Jia-Li Pu, Bao-Rong Zhang

**Affiliations:** 1 Department of Neurology, Second Affiliated Hospital, School of Medicine, Zhejiang University, Hangzhou 310009, Zhejiang, China; 2 Department of Anesthesiology, Women's Hospital, School of Medicine, Zhejiang University, Hangzhou 310009, Zhejiang, China; 3 Department of Neurology, Affiliated Hangzhou First People’s Hospital, Zhejiang University School of Medicine, Hangzhou 310006, Zhejiang, China

**Keywords:** Parkinson's disease, α-synuclein, brain perivascular space, glymphatic system

## Abstract

Perivascular spaces in the brain have been known to communicate with cerebrospinal fluid and contribute to waste clearance in animal models. In this study, we sought to determine the association between MRI-visible enlarged perivascular spaces (EPVS) and disease markers in Parkinson’s disease (PD). We obtained longitudinal data from 245 patients with PD and 98 healthy controls from the Parkinson’s Progression Marker Initiative. Two trained neurologists performed visual ratings on T2-weighted images to characterize EPVS in the centrum semiovale (CSO), the basal ganglia (BG) and the midbrain. We found that a greater proportion of patients with PD had low grade BG-EPVS relative to healthy controls. In patients with PD, lower grade of BG-EPVS and CSO-EPVS predicted lower CSF α-synuclein and t-tau. Lower grade of BG-EPVS were also associated with accelerated Hoehn &Yahr stage progression in patients with baseline stage 1. BG-EPVS might be a valuable predictor of disease progression.

## INTRODUCTION

Perivascular spaces (PVS), also known as Virchow-Robin spaces, are fluid-filled cavities that surround the perforating vessels in the brain. MRI-visible enlarged perivascular spaces (EPVS) have been recognized as a hallmark of small vessel disease [1], and commonly seen in the elderly. The emerging notion of the glymphatic system suggests that PVS are an essential component of a glial-dependent para-vascular extracellular fluid circulation system that facilitates brain waste clearance [2, 3]. Several lines of evidence suggested that PVS participates in the clearance of Aβ, and its dysfunction partially contributes to the deposition of Aβ [4–6]. In addition, PVS enlargement is associated with accumulation of blood-derived substances such as fibrin and subsequent neurotoxicity in an animal model of blood-brain barrier (BBB) dysfunction [7]. These findings suggest that PVS changes might be pathologically linked to abnormal protein aggregation. However, it has not been solidly established in human as to whether MRI-visible EPVS are associated with abnormal protein accumulation in neurodegenerative disorders.

Parkinson’s disease is a neurodegenerative disorder that is pathologically characterized by aggregation of misfolded α-synuclein and loss of dopaminergic neurons [8]. Atypical, asymmetric giant EPVS has been reported in patients with PD [9–11]; however, few studies have examined the role typical EPVS plays in PD. One study in a small cohort of patients with PD found a correlation between EPVS and global cognitive performances [12]. However, recent evidence suggested that PVS and the glymphatic system also contributed to the clearance of α-synuclein [13]. In addition, increased BBB permeability and fibrin accumulation in the PVS has been reported in the striatum of postmortem PD brain, although PVS area was not quantitatively measured in autopsy [14]. For these reasons, further research regarding the clinical implications of EPVS in PD is warranted.

In the present study, using data from the Parkinson’s Progression Marker Initiative (PPMI), we explored the link between MRI-visible EPVS and CSF biomarkers in patients with PD and healthy controls (HC). We also explored the clinical significance of EPVS in relation to motor and nonmotor symptom progression in patients with PD.

## RESULTS

### Baseline characteristics and EPVS in PD patients and HC

The final analysis included 245 patients with PD and 98 HCs. Baseline characteristics are presented in Table 1. Notably, patients with PD had a higher percentage of low-grade EPVS in the BG. Although the percentage of smoking history and diabetes mellitus was statistically different between patients with PD and HC, these two factors were not associated with the burden of EPVS according to previous studies [15, 16]

**Table 1:** Baseline characteristics and EPVS of patients with PD and healthy controls.

	**PD, n=245**	**HC, n=98**	**p value**
**Demographic characteristics**			
Age,^a^ y, mean (SD)	60.60 ± 9.83	60.58 ± 10.70	0.935
Male, ^b^ n (%)	155 (63.2)	66 (67.3)	0.476
Education,^a^ y, mean (SD)	15.31 ± 2.96	15.99 ± 2.72	0.081
Race, White, ^b^ n (%)	227 (92.6)	91 (92.8)	0.948
APOE e4 carriers, ^b^ n (%)	62 (25.3)	21 (21.4)	0.401
missing	21	7	
Smoking history, ^b^ n (%)	74 (30.2)	41 (41.8)	0.039
Age at disease onset, y, mean (SD)	58.45 ± 10.20	-	
Disease duration, m, mean (SD)	6.60 ± 6.75	-	
**Vital signs, mean (SD)**
Supine systolic blood pressure, ^a^ mmHg	132.04 ± 17.01	133.00 ± 16.38	0.554
Heart rate, ^a^ bpm	67.72 ± 10.88	66.48 ± 9.40	0.283
BMI, ^a^ kg/m^2	27.13 ± 4.60	27.00 ± 4.60	0.877
**Medical History**			
Hypertension, ^b^ n (%)	76 (31.0)	36 (36.7)	0.308
Diabetes mellitus, ^b^ n (%)	23 (9.3)	2 (5.0)	0.043
missing	4	0	
Dyslipidemia, ^b^ n (%)	90 (36.7)	17 (42.5)	0.116
**Clinical Evaluation, mean (SD)**
MDS-UPDRS III^a^	20.34 ± 8.82	0.76 ± 1.81	0.000
MOCA^a^	27.31 ± 2.22	28.33 ± 1.17	0.000
missing	3	0	
			
GDS_15^a^	2.22 ± 2.27	1.19 ± 2.20	0.000
			
STAI^a^	64.38 ± 17.70	56.60 ± 13.74	0.000
			
**EPVS CSO**			
low (score ≤2), ^b^ n (%)			
high (score ≥3), ^b^ n (%)	141 (57.6)	50 (51.0)	0.271
**BG**	104 (42.4)	48 (49.0)	
low (score ≤1), ^b^ n (%)			
high (score ≥2), ^b^ n (%)	177 (72.2)	52 (53.1)	0.001
**Midbrain**	68 (27.8)	46 (46.9)	
No (score 0), ^b^ n (%)			
Yes (score 1), ^b^ n (%)	67 (27.3)	22 (22.4)	0.350
	178 (72.6)	76 (77.5)	

PD, Parkinson’s disease; BMI, Body Mass Index; MDS-UPDRS, Movement Disorder Society-Unified Parkinson’s Disease Rating Scale; MoCA, Montreal Cognitive Assessment; STAI, State-Trait Anxiety Inventory; GDS_15, Geriatric Depression Scale Short_15; INQ, interquartile range; EPVS, enlarged perivascular spaces; CSO, centrum semiovale; BG, basal ganglia.

^a^Mann-Whitney U tests.

^b^χ2 test.

### EPVS and biomarkers

**Figure 1:**
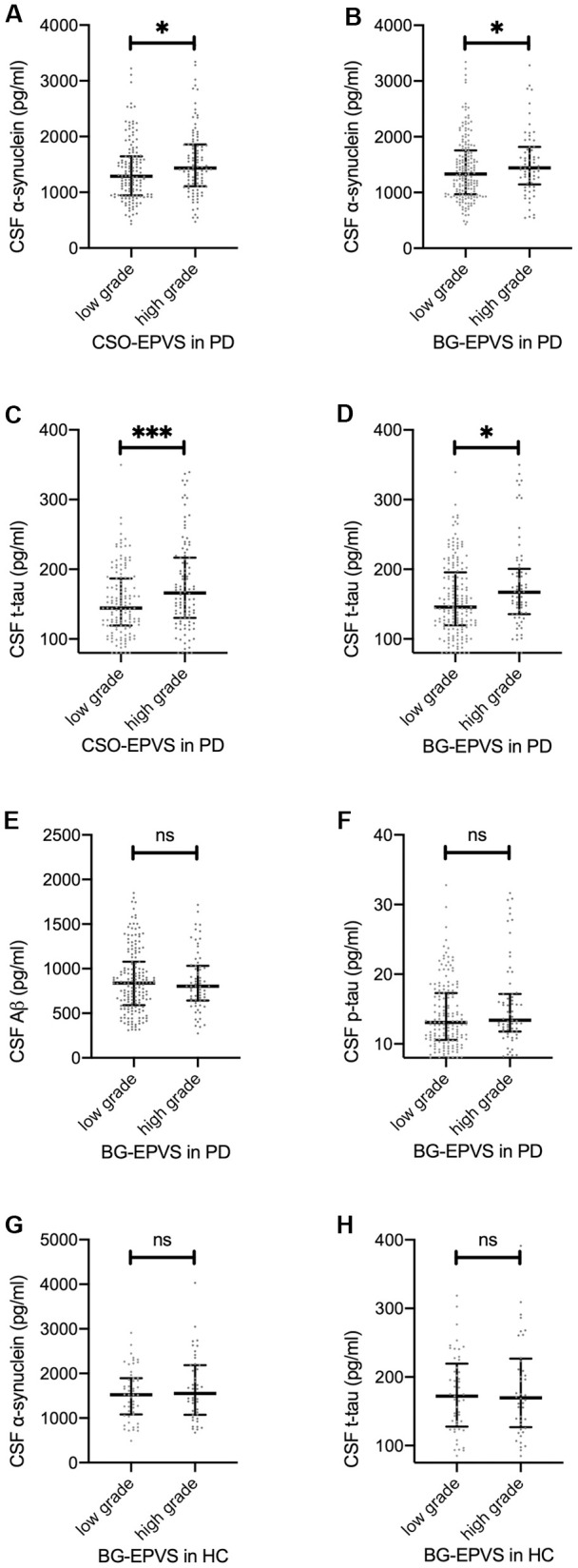
**Association between EPVS and CSF proteins.** (**A–D**): baseline CSF α-synuclein (**A**, **B**) and t-tau (**C**, **D**) values separated by low/high grade CSO EPVS (**A**, **C**) and BG EPVS (**B**, **D**) in patients with PD. (**E**, **F**): baseline CSF Aβ (E) and p-tau (**F**) values separated by low/high grade BG-EPVS in patients with PD. (**G**, **H**): baseline CSF α-synuclein (**G**) and t-tau (H) values separated by low/high grade BG-EPVS in patients with PD. Lines represent median with interquartile range. Significance on figures represent Spearman correlation of baseline EPVS and CSF proteins. For result of repeated measure linear mixed model, please refer to Table 2.

Overall, patients with lower EPVS scores had lower CSF protein values. After adjusting for age and sex, there were significant main effects of CSO-EPVS and BG-EPVS scores on CSF α-synuclein and t-tau levels (Table 2 and Figure 1A–1D), but not on p-tau nor Aβ levels (Figure 1E, 1F) in the mixed model. After cases with CSF hemoglobin levels > 200 ng/ml were excluded (N = 141), the effect of BG-EPVS remained significant, and the effect of CSO-EPVS became marginally significant (Table 2).

**Table 2:** Association between CSF biomarkers and EPVS.

	**CSO-EPVS**		**BG-EPVS**		**Midbrain-EPVS**	
	**Estimate (95% CI)**	**p**	**Estimate (95% CI)**	**p**	**Estimate (95% CI)**	**p**
**CSF α-synuclein**	98.6 (3.1, 194.0)	0.044	138.2 (6.7, 269.7)	0.041	29.7 (-137.9, 197.3)	0.729
**CSF α-synuclein (Low Hb)**^a^	92.1 (-9.7, 193.7)	0.076	180.3 (40.7, 320.0)	0.012	47.8 (-130.4, 226.0)	0.528
**CSF t-tau**	10.5 (1.5, 19.5)	0.023	12.8 (0.4, 25.2)	0.045	-2.1 (-18.0, 13.8)	0.796
**CSF p-tau**	0.78 (-0.16, 1.71)	0.104	0.73 (-0.49, 1.95)	0.240	-0.79 (-2.33, 0.75)	0.316
**CSF Aβ**	25.3 (-33.1, 86.6)	0.397	24.1 (-57.5, 105.8)	0.563	-27.0 (-127.7, 73.7)	0.600

Note: Since there was no significant effect of disease duration * EPVS interaction, values in this table were derived from linear mixed model that did not contain the interaction as a fixed effect.

^a^ Cases with CSF hemoglobin levels > 200 ng/ml were eliminated (N=141 across time).

The association between EPVS and CSF protein at baseline is shown in Figure 1. For HCs, no significant associations between CSF protein values and EPVS scores were observed (Figure 1G, 1H).

### EPVS, motor symptoms and disease severity

**Figure 2:**
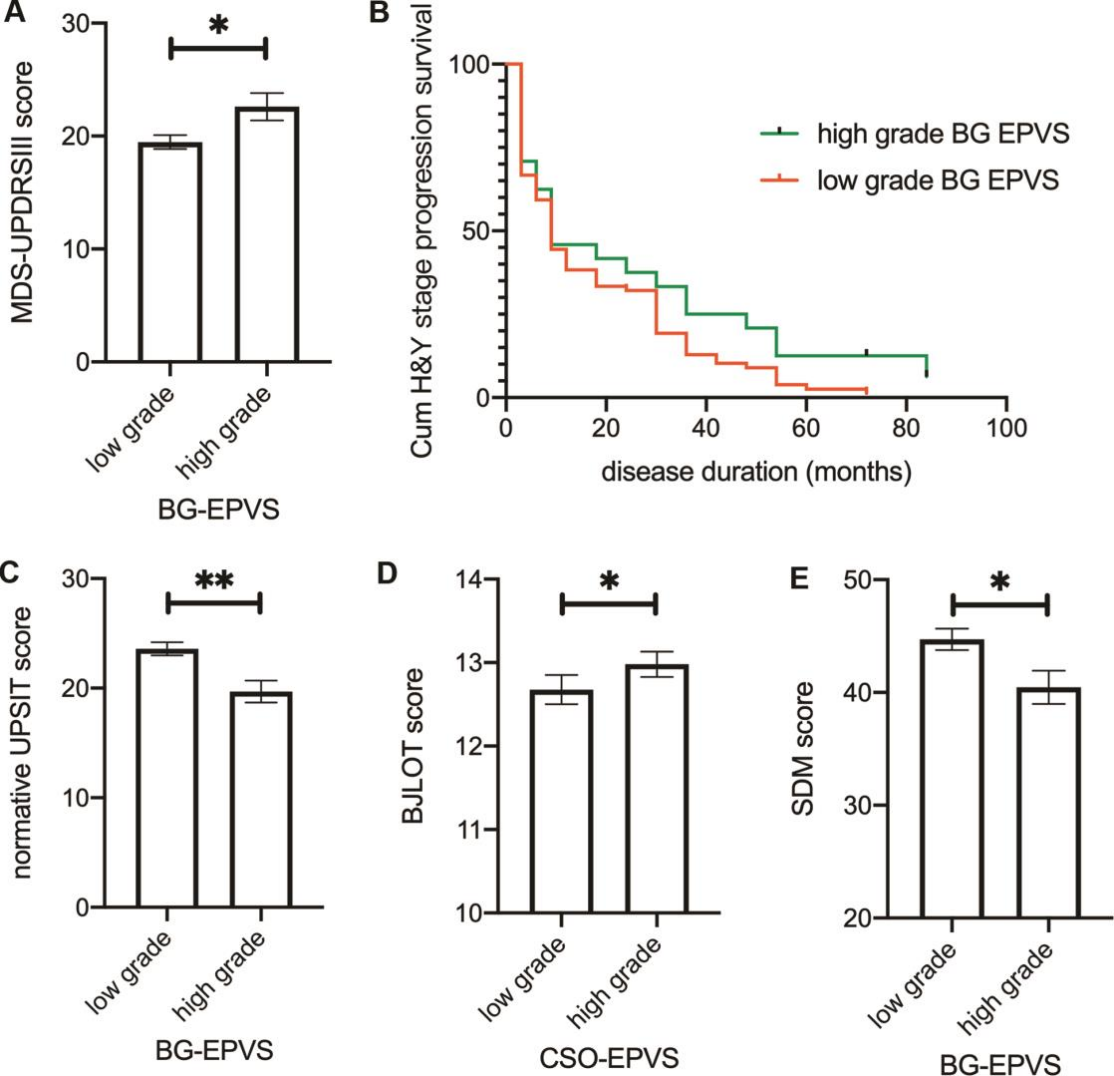
**Association between EPVS and clinical outcomes.** (**A**) Baseline MDS-UPDRS III score separated by BG-EPVS grades. (**B**) Kaplan–Meier estimation of time to transit from baseline H&Y grade 1 to grade 2 between low-grade and high-grade BG-EPVS groups. Patients with low-grade BG-EPVS had increased risk of H&Y stage progression (HR: 0.637, 95% CI: 0.411-0.986, P=0.033) compared to patients with high-grade BG-EPVS. (**C-E**) Baseline normative UPSIT score (**C**), BJLOT score (**D**), and SDM score (**E**) separated by EPVS grades in BG (**C, E**) and CSO (**D**). Note that there were no significant time*EPVS interaction effects, significance on figures are calculated from multivariate regression using baseline data. For result of repeated measure linear mixed model, please refer to table 2. Lines represent mean ± SEM. UPSIT: University of Pennsylvania Smell Identification. Test BJLOT: Benton Judgment of Line Orientation Test SDM: Symbol Digit Modality Test.

Neither EPVS score nor EPVS score by disease duration interaction effect had a significant effect on MDS-UPDRS III scores in the linear mixed model. However, we found that higher BG-EPVS scores were associated with higher baseline MDS-UPDRS III scores after adjusted for age of disease onset and sex in a linear regression model (β = 2.094, 95% CI: 0.152–4.036, p = 0.036) (Figure 2A).

The loss-to-follow-up rate after year 3 was 1.87% (2/107) for patients with baseline H&Y stage 1 and 11.76% (16/136) for stage 2 patients. For H&Y stage 1 patients at baseline (N = 107), Cox regression revealed a decreased risk of H&Y stage progression in patients with low-grade BG-EPVS (HR = 0.623, 95% CI: 0.402 - 0.963, P = 0.033) after adjusted for baseline MDS-UPDRS III score, sex, age at diagnosis, and levodopa equivalent doses. There was no such effect for stage 2 patients at baseline (N = 136, HR = 1.204, 95% CI: 0.657–2.206).

In brief, we found that higher BG-EPVS levels were associated with higher MDS-UPDRS III scores at baseline. Lower BG-EPVS levels were associated with faster H&Y stage progression.

### EPVS, nonmotor symptoms, and dopaminergic innervation

The associations between EPVS and nonmotor symptoms are presented in Table 3. We found that high BG-EPVS were associated with low normative UPSIT scores and symbol digit modalities scores, which represent olfactory function and attention-processing speed, respectively (Figure 2C, 2E). Additionally, we observed a mildly elevated score on the Benton Judgment of Line Orientation, a visuospatial perception test, in patients with high CSO-EPVS scores (Figure 2D). We observed no significant associations between EPVS and global cognition, memory, working memory, or executive function, and striatal binding ratio. The EPVS score * disease duration interaction effect was not significant in any of the models.

**Table 3:** Association between clinical variables and EPVS.

	**CSO-EPVS**		**BG-EPVS**		**Midbrain-EPVS**	
	**Estimate (95% CI)**	**p**	**Estimate (95% CI)**	**p**	**Estimate (95% CI)**	**p**
**MDS-UPDRS I**	-0.01 (-0.63, 0.61)	0.972	0.23 (-0.63, 1.07)	0.606	-0.19 (-1.27, 0.89)	0.728
**MDS-UPDRS II**	-0.02 (-0.76, 0.73)	0.970	0.57 (-0.47, 1.60)	0.282	0.91 (-0.40, 2.21)	0.174
**MDS-UPDRS III**	-0.46 (-1.92, 1.00)	0.538	0.49 (-1.52, 2.51)	0.632	-0.10 (-2.65, 2.45)	0.940
**UPSIT**	-0.74 (-2.00, 0.51)	0.248	-2.08 (-3.86, -0.29)	0.023	-1.93 (-4.11, 0.25)	0.085
**Mean caudate SBR**	-0.05 (-0.14, 0.03)	0.230	0.05 (-0.07, 0.16)	0.449	-0.06 (-0.21, 0.08)	0.393
**Mean putamen SBR**	0.00 (-0.04, 0.04)	0.864	0.02 (-0.04, 0.08)	0.470	-0.04 (-0.12, 0.03)	0.246
**SCOPA**	0.48 (-0.81, 1.77)	0.466	1.16 (-0.63, 2.94)	0.205	1.57 (-0.67, 3.81)	0.171
**MoCA**	0.15 (-0.24, 0.54)	0.442	0.06 (-0.50, 0.62)	0.825	0.55 (-0.11, 1.22)	0.106
**BJLOT**	0.39 (0.11, 0.67)	0.007	0.06 (-0.35, 0.46)	0.786	0.42 (-0.07, 0.90)	0.094
**HVLT total recall**	-0.10 (-1.73, 1.53)	0.907	-0.84 (-3.15, 1.49)	0.481	1.94 (-0.86, 4.73)	0.176
**HVLT delayed recall**	-0.34 (-1.97, 1.29)	0.682	-1.05 (-3.37, 1.28)	0.380	1.08 (-1.73, 3.89)	0.451
**HVLT retention**	-0.43 (-1.65, 0.79)	0.493	-1.17 (-2.93, 0.61)	0.199	-0.09 (-2.21, 2.02)	0.932
**HVLT rec disc index**	-0.46 (-1.82, 0.90)	0.509	-0.23 (-2.20, 1.74)	0.819	1.64 (0.71, 3.99)	0.172
**LNS**	-0.06 (-0.45, 0.34)	0.779	-0.18 (-0.74, 0.38)	0.530	0.37 (-0.31, 1.04)	0.288
**Semantic Fluency Test**	1.06 (-0.46, 2.58)	0.173	0.12 (-2.05, 2.29)	0.915	0.05 (-2.58, 2.67)	0.973
**Symbol Digit Modalities**	-1.15 (-2.59, 0.28)	0.117	-2.03 (-4.05, -0, 01)	0.050	-0.68 (-3.20, 1.84)	0.599

MDS-UPDRS: Movement Disorder Society-Unified Parkinson's Disease Rating Scale

UPSIT: University of Pennsylvania Smell Identification Test.

SBR: Striatal Binding Ratio.

SCOPA: Scales for Outcome in Parkinson's Disease.

MoCA: Montreal Cognitive Assessment.

BJLOT: Benton Judgment of Line Orientation Test.

HVLT: Hopkins Verbal Learning Test.

HVLT Rec disc index: Hopkins Verbal Learning Test Recognition discrimination index.

LNS: Letter Number Sequencing.

## DISCUSSION

Inspired by recent findings that PVS are involved in brain waste clearance and that there may be a bidirectional relationship between dysfunction in this system and neurodegenerative disorders [17], the current study examined the relationship between MRI-visible EPVS and disease markers. We found that the proportion of low-grade BG-EPVS was higher in patients with PD than in HCs. Lower BG-EPVS and CSO-EPVS scores were associated with decreased CSF α-synuclein and t-tau values. In addition, lower BG-EPVS score was associated with accelerated H&Y stage progression.

Counterintuitively, we found that a lower portion of patients with PD had high-grade BG-EPVS relative to HCs. This finding was in line with one recent study (in preprint) that computationally measured PVS volume fraction in patients with early cognitive decline [18]. The authors found a significantly lower PVS volume fraction in the anterosuperior medial temporal lobe, which is primarily involved in early stage Alzheimer’s disease. These findings suggested that the loss of observable signals of EPVS might be a manifestation of dysfunctional perivascular flow caused by abnormal protein aggregation, because PVS visibility on MRI relies strictly on CSF signal detection. In PD, it is likely that there is involvement of misfolded α-synuclein. This was supported by the finding that lower EPVS levels correlate with lower CSF α-synuclein values in the current study, which were attributable to increased aggregation of α-synuclein within brain [19]. It is also likely that abnormal proteins (e.g., fibrin) that are associated with increased BBB permeability in striatum [14] are involved. However, it should be noted that the potential association between EPVS and protein accumulation is likely region-specific, and EPVS in other regions are likely associated with risk factors of cerebral small vessel disease as established in previous studies [20].

Contrary to previous theories that MRI-visible EPVS represent waste accumulation and predict worse clinical outcomes [21], we found that lower BG-EPVS levels are associated with decreased CSF α-synuclein, t-tau, and accelerated H&Y progression. Previous studies have established that there is decreased CSF α-synuclein levels in patients with PD [22, 23], and although controversial [24, 25], low CSF α-synuclein appears to predict increased disease severity [23, 26–28]. In general, t-tau and p-tau levels were closely correlated. However, t-tau was recognized as a nonspecific marker of neuronal injury and degenerative changes [29], while p-tau specifically represents tau pathology [30]. As levels of p-tau and Aβ did not differ between EPVS levels in patients with PD, and the effects of EPVS on CSF proteins were not replicated in HCs, the current findings indicated disease-specific PVS dysfunction in patients with PD.

Evidence linking low grade BG-EPVS to worse clinical outcomes in this study included the decreased risk of H&Y progression in baseline stage 1 patients revealed by Cox regression, which concords with previous reports regarding an association between decreased CSF and disease severity [23, 26, 27]. However, several other observations in this study do not support this association. First, there was no association between BG-EPVS and dopaminergic innervation as reflected by the striatal binding ratio quantified using DaT scan. However, a recent post-mortem study reported that neuron counts in the substantia nigra pars compacta did not correlate with mean striatal binding ratio [31]. Second, we did not find a significant effect of BG-EPVS on MDS-UPDRS score progression across time. This was partially explained by the fact that the H & Y scale was more responsive to progression than the UPDRS III [32]. Third, Cox regression for baseline H&Y stage 2 patients did not yield a consistent result. We would argue that the much higher loss-to-follow-up rate in stage 2 patients caused a bias. The loss-to-follow-up rate after year 3 was 1.87% in baseline H&Y stage 1, while this number was 11.76% in stage 2, exceeding 5%, and potentially threatening the validity of the data [33]. Therefore, the potential association between BG-EPVS and disease progression remains to be verified in further studies.

In the current study, EPVS levels in both CSO and BG correlated with CSF α-synuclein and t-tau levels, while low BG EPVS levels were more prevalent in PD and were associated with accelerated H&Y stage. It is likely that, in regions that were not primarily involved with abnormal protein accumulation, EPVS represents an increased secretion of water/decreased clearance of fluid, and/or increased resistance to perivascular fluid flow that leads to dilution of CSF proteins.

Although previously considered benign in elderly adults, several studies have reported negative associations between EPVS scores and cognitive function in healthy participants [34, 35] and in patients with previous cerebrovascular incidents [36]. In this cohort of patients with PD, we found that higher BG-EPVS scores were associated with worse olfactory function and attention-processing speed. Olfactory dysfunction is one of the prodromal symptoms of PD [37]. A recent longitudinal study revealed that progressive olfactory impairment during the course of PD was associated with basal ganglia volume loss [38]. Our finding that BG-EPVS was negatively associated with attention-processing speed is accordance with previous studies in patients with cerebral small vessel disease [39] and the general population [40]. In addition, several cognitive psychology studies have demonstrated that BG plays a crucial role in attention shifting [41, 42]. Taken together, it appears that high grade EPVS distort the normal anatomy, destroying neighboring white matter tracts, and subsequently causing reduced performance in associated neural processes.

In the current study, we incidentally found that patients with higher CSO-EPVS scores had mildly better visuospatial task performance. One recent meta-analysis revealed a similar finding in that EPVS in the hippocampus contributed to better memory performances [36]. These findings require further validation, and the mysterious nature of the positive association between EPVS and cognitive performances remains to be determined.

This study has some limitations. First, in the current study, we did not find any associations between midbrain EPVS and biomarkers. It is likely that the binary rating (0 for none vs. 1 for ≥ 1 EPVS counted) in the slice of midbrain with highest number of EPVS [43] potentially underestimates the effects of midbrain EPVS. As MRI slice thickness (2, 3, 4, 5 mm) varies across different sites, attempts to summarize a total midbrain burden across levels would cause a bias in such a cohort. Midbrain is primarily involved with α-synuclein deposition in PD [44], yet midbrain EPVS received less attention relative to EPVS in CSO and BG in the literature. The underlying pathophysiology of midbrain EPVS and its potential association with neurodegenerative diseases, PD in particular, remains to be further studied. Second, future studies that explore the relative contributions of BBB permeability and glymphatic system dysfunction to the EPVS and protein aggregation by contrast MRI would be necessary. Third, as a limited number of patients had MRI scans over the follow-up period, we did not examine the dynamic relationship between changes in EPVS and CSF biomarkers and clinical variables. Besides, for such a study, it would be necessary to examine the replicability of the current findings in other cohorts and to calculate EPVS by automated, computational quantification methods. In addition, further studies that explore the region-specific roles of EPVS in neurodegenerative diseases may be necessary. Future studies could utilize PET amyloid and tau imaging, and computationally calculate the volume of EPVS in key regions of interest in MRI images.

Extending the previous understanding that BG-EPVS was associated with hypertensive arteriopathy-related intracerebral hemorrhage [45, 46], the current findings suggest that BG-EPVS also likely played a part in neurodegenerative disorders. Our results suggest that, MRI-visible EPVS have a complex association with disease factors in patients with PD. On the one hand, decreased visibility of EPVS in key regions, as a potential consequence of obstructed CSF flow in PVS, were likely related to clinical events that were the consequence of abnormal protein aggregation (e.g., disease stage progression). On the other hand, a high EPVS burden likely resulted in disrupted local neuroanatomy related to worsening of specific symptoms such as olfaction, and attention-processing speed.

## MATERIALS AND METHODS

### Data sources

The data for this study were obtained from the Parkinson’s Progression Marker Initiative (PPMI) [47], which is an ongoing, prospective, longitudinal, observational, international, and multicenter study aimed at identifying PD biomarkers. We obtained approval to access the PPMI database and investigated CSF, clinical, and neuroimaging data. The PPMI study was approved by the institutional review board of all participating sites, and written informed consent was obtained from all participants by the site investigators.

### Study participants

Major inclusion criteria for the PD cohort in the PPMI included were as follows: (1) drug naïve; (2) diagnosed with PD within the past 2 years; (3) Hoehn and Yahr (H and Y) stage 1 or 2 at baseline; (4) age 30 years or older; and (5) striatal dopaminergic dysfunction on SPECT. Of the whole PPMI-PD cohort, a total of 287 patients with PD and 129 HC had baseline CSF protein values measured and underwent a T2 MRI scan. Of these, 42 patients and 31 HCs were excluded from this study based on the following exclusion criteria: (1) extensive concomitant white matter hyperintensity in the CSO that hindered the visibility of EPVS; (2) ischemic stroke at baseline or follow-up, which potentially alters the natural clinical course of PD; (3) poor image quality (low spatial resolution, poor image contrast, artifacts) that caused difficulties in rating; and (4) incomplete baseline demographic information. Finally, 245 patients with PD and 98 HCs were included in the final analysis. There were no significant difference between patients included and patients excluded in terms of demographics, clinical evaluation scales or CSF protein values, except for a mildly shorter duration (included: 6.6 ± 6.7 months; excluded: 8.1 ± 6.8 months; p = 0.025) and lower baseline Movement Disorder Society-sponsored revision of the Unified Parkinson's Disease Rating Scale III (MDS-UPDRS III) score (included: 20.3 ± 8.8; excluded: 23.7 ± 9.9; p = 0.021). Clinical features, including demographic characteristics, motor severity, cognitive testing, and biomarkers were systematically assessed according to the PPMI study protocol.

### Clinical assessment

Demographic and clinical features assessed for each patient included age, sex, years of education, race, smoking history, apolipoprotein E (APOE) e4 allele carriage state, body mass index, and metabolic indices.

Motor symptoms were evaluated at baseline, 3, 6, 9, 12, 18, 24, 30, 36, 42, 48, 54, 60, 72, 84, and 96 months. Rating scales included the MDS-UPDRS, the Modified Schwab and England Activities of Daily Living Scale, and H&Y staging. We analyzed UPDRS scores that were rated during the off condition (levodopa/dopaminergic agonist withheld for at least 6 hours prior to the visit).

Nonmotor symptoms were evaluated on a yearly basis. Cognitive tests included the Montreal Cognitive Assessment for global cognition, the Hopkins Verbal Learning Test for memory, the Benton Judgment of Line Orientation Test for visuospatial perception, the Letter-Number Sequencing and Semantic Fluency Test for working memory and executive function, and the Symbol Digit Modality Test for attention- processing speed. Olfaction was assessed using the University of Pennsylvania Smell ID test at baseline and was not evaluated annually. Autonomic function was evaluated using the Scale for Outcomes in Parkinson's Disease-Autonomic Dysfunction.

### CSF sample collection and analysis

CSF was collected at baseline, 6, 12, 24, and 36 months. Levels of CSF Aβ42, total tau (t-tau), and phosphorylated tau (p-tau) at threonine 181 position were measured using the xMAP-Luminex platform with INNOBIA AlzBio3 immunoassay kit-based reagents. CSF α-synuclein concentrations were analyzed using commercially available enzyme-linked immunosorbent assay kits.

### Dopamine transporter imaging

Dopamine imaging was performed by DaTscan at baseline, 12, 24, and 48 months. Quantitative DaTscan measures in striatal binding ratio of caudate and putamen uptake were downloaded.

### MRI acquisition

According to PPMI protocol, Non-contrast enhanced T2-weighted brain MRI images were acquired using 1.5 or 3 Tesla MRI scanners at baseline. The T2-weighted images (axial acquisition, total scan time of 5 min and 8 s) were obtained from 21 different centers as part of the PPMI database. To minimize data bias between sites, the PPMI core optimized the acquisition sequence acquisition parameters across sites to maximize data comparability. For a detailed description of MRI protocols, please refer to ppmi-info.org.

### Manual rating of MRI-visible EPVS

**Figure 3:**
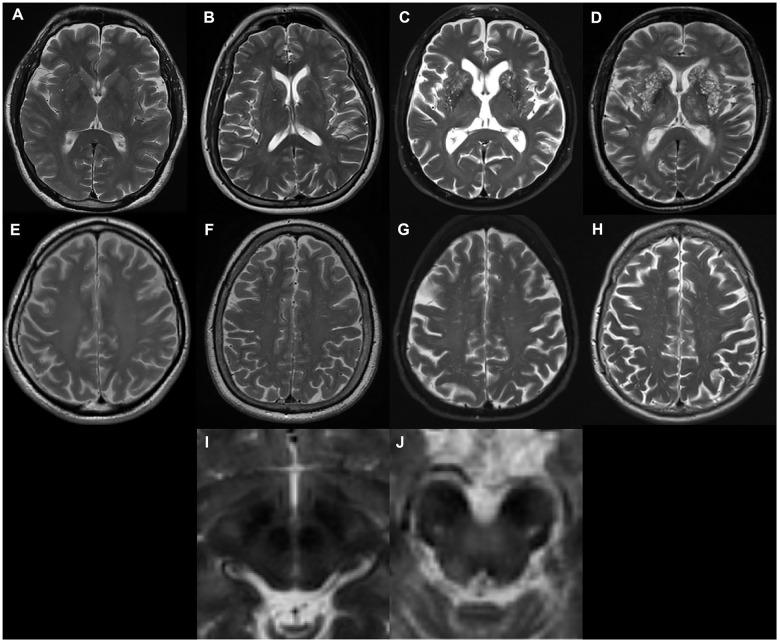
**Examples of MRI-visible perivascular spaces.** (**A–D**) Examples of MRI-visible BG-EPVS. (A) grade 1; (**B**) grade 2; (**C**) grade 3; (**D**) grade 4. (**E–H**) Examples of MRI-visible CSO-EPVS. (**E**) grade 1; (**F**) grade 2; (**G**) grade 3; (**H**) grade 4. (**I**) Example of no MRI-visible midbrain-EPVS. (**J**) Examples of MRI-visible midbrain-EPVS.

EPVS were defined as fluid-filled spaces with a signal intensity similar to CSF on all sequences, which followed the course of penetrating vessels, and were linear, round, or ovoid in shape. Following the STandards for ReportIng Vascular changes on nEuroimaging (STRIVE) [1], two trained neurologists (L.Y.G. and J.T. with 2-years and 9-years of experience, respectively), who were blinded to subject’s clinical information, manually counted the numbers of EPVS independently. According to well-established PVS rating criteria [43], EPVS were rated using a 4-point visual rating scale on axial-T2 weighted images (0: no EPVS; 1: <10 EPVS; 2: 11–20 EPVS; 3: 21–40 EPVS; and 4: >40 EPVS) for the CSO and BG. Midbrain EPVS were rated in a binary fashion (0 for no EPVS, 1 for EPVS visible). EPVS rating was carried out on the slice with the highest number of EPVS after all the relevant slices for each anatomic area were scanned. Both hemispheres were counted, and the hemisphere with the higher score was used if there was asymmetry between the sides [43]. 'Representative figures of EPVS rating are demonstrated in Figure 3. As a limited number of patients had EPVS scores of 0 or 4, CSO-EPVS scores were further categorized into low-grade (score ≤ 2) and high-grade (score ≥ 3) and BG-EPVS scores were categorized into low-grade (score ≤ 1) and high-grade (score ≥ 2) for illustrative purposes. These categorizations are demonstrated in the figures; however, statistical analysis was carried out using the original values. Discrepancies were solved by consensus. T2-weighted images acquired at baseline were preferentially rated. In a few cases when images were not acquired at baseline, images acquired at follow-up visits were rated. The inter-rater Cohen’s kappa scores were 0.63, 0.75, and 0.96 for the CSO, the BG and the midbrain EPVS scores, respectively. Major causes of discrepancies in EPVS scores included EPVS numbers at borderline, and vague lines.

### Statistical analysis

Data were downloaded from the PPMI database on April 2020. Statistical analysis was performed using SPSS software (IBM Corp, Armonk NY, USA). Graphs were plotted using GraphPad (San Diego, CA, USA). Demographic data, CSF biomarkers, clinical variables were compared between groups (PD patients included vs PD patients excluded and PD patients included vs HC included) using Chi-square tests for categorical variables and Mann-Whitney U tests for continuous variables.

Repeated-measure linear mixed models with random intercept were used to examine the effects of EPVS across time. We incorporate patients as random effects. Fixed effects included EPVS score, age, sex, and disease duration (years). For cognitive tests, we added apoe4 carrier status (positive: e2/e4, e3/e4, e4/e4 or negative: e2/e2, e2/e3, e3/e3) and years of education. Interactions between EPVS score and disease duration were treated as fixed effects in separate models to investigate whether EPVS score was associated with slope changes. For the statistical models we used initial EPVS scores. For illustrative purposes, we depicted high/low levels for BG- and CSO-EPVS, due to the low number of cases in some of the score groups. For variables that had a significant main effect of EPVS, but no EPVS * disease duration interaction, we plotted their values at baseline for illustrative purposes.

Using the same standard in a previous study that assessed longitudinal changes in CSF biomarkers in the PPMI cohort, three outliers of CSF α-synuclein levels that exceeded the 95% CI were excluded from the analysis [25]. CSF t-tau and p-tau levels that were recorded as < 80 pg/ml and < 8 pg/ml were excluded from the mixed model. To minimize the effect of CSF hemoglobin hemolysis on CSF α-synuclein, we also reported results after samples with CSF hemoglobin >200 ng/ml were excluded. The effect of EPVS on baseline UPSIT was tested using linear regression, since no longitudinal data was available. Covariates include age and sex.

A Cox regression was performed to examine the association between EPVS scores and H&Y stage progression. Age of disease onset, sex, baseline MDS-UPDRS III score were entered as covariates. Separate survival analysis was conducted for PD patients with baseline H&Y stage at 1 and 2. Covariates included baseline MDS-UPDRS III score, sex, age at diagnosis, and levodopa equivalent doses.

Values with p<0.05 were regarded as statistically significant.
